# Supplementary material for: Highly Efficient Cpf1-Mediated Gene Targeting in Mice Following High Concentration Pronuclear Injection
Source: G3 (Bethesda). 2016 Dec 30;7(2):719–22. doi: 10.1534/g3.116.038091 (PMC5295614; doi:10.1534/g3.116.038091)
Supplement: Supplementary file 1 [file 719FigureS1.pdf]

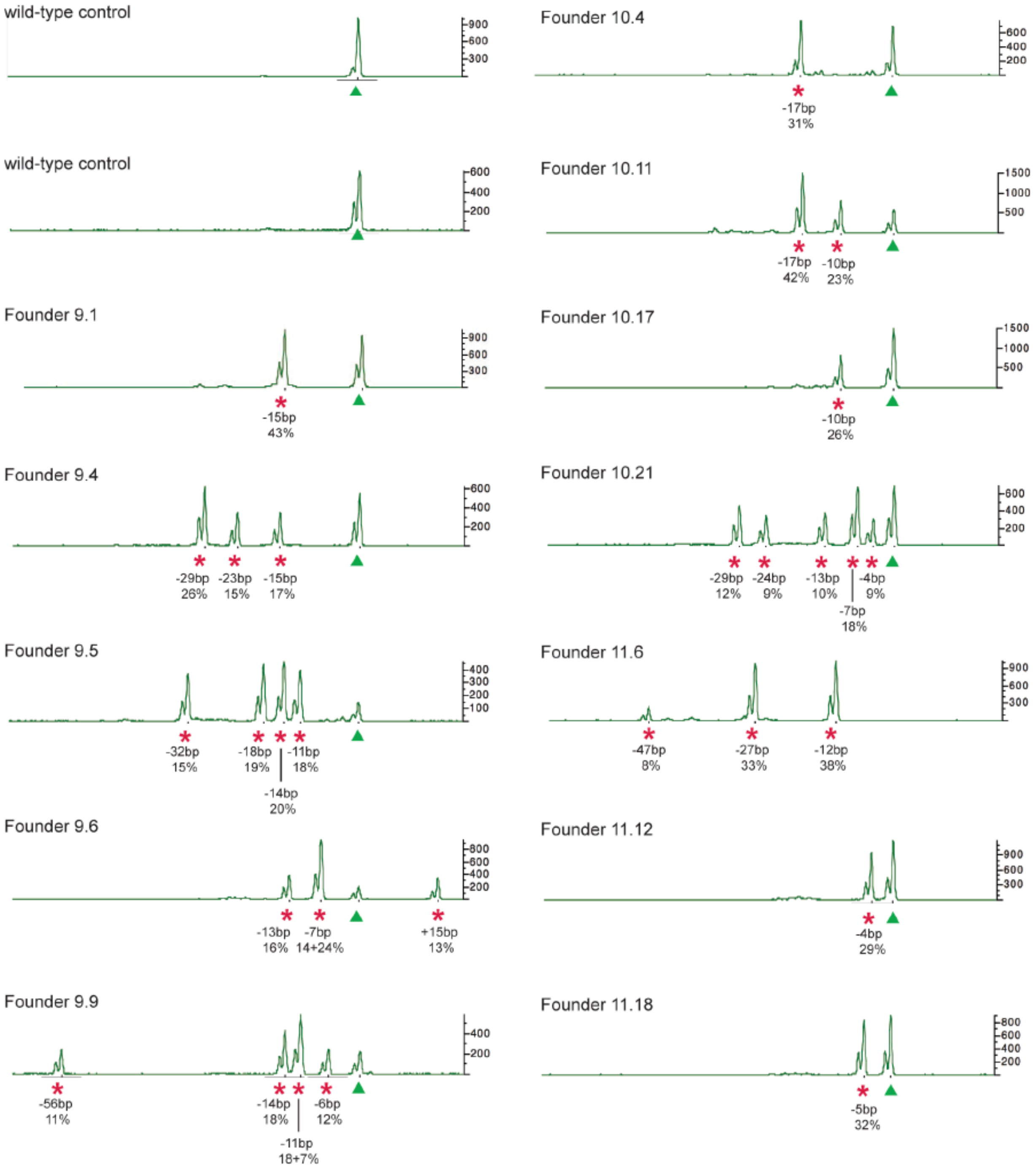

Figure S1 Examples of 2 wild-type and 12 founder mice screened with a fluorescently labeled PCR product. WT *Tyr* allele products are indicated (green arrowhead) as well as mutant *Tyr* alleles confirmed by deep sequencing of an independent PCR product (red *). For mutant alleles, the size of each allele relative to wild-type is indicated along with the percentage of sequencing reads. Note that founder 11.6 lacks a wild-type allele. Upon sequencing, we determined that the single -7bp peak in founder 9.6 was actually a mix of two different -7bp mutant alleles. Similarly, the -11bp peak in founder 9.9 contained two different -11bp mutant alleles.
