# Supplementary material for: Highly Efficient Cpf1-Mediated Gene Targeting in Mice Following High Concentration Pronuclear Injection
Source: G3 (Bethesda). 2016 Dec 30;7(2):719–22. doi: 10.1534/g3.116.038091 (PMC5295614; doi:10.1534/g3.116.038091)
Supplement: Supplementary file 2 [file 719FigureS2.pdf]

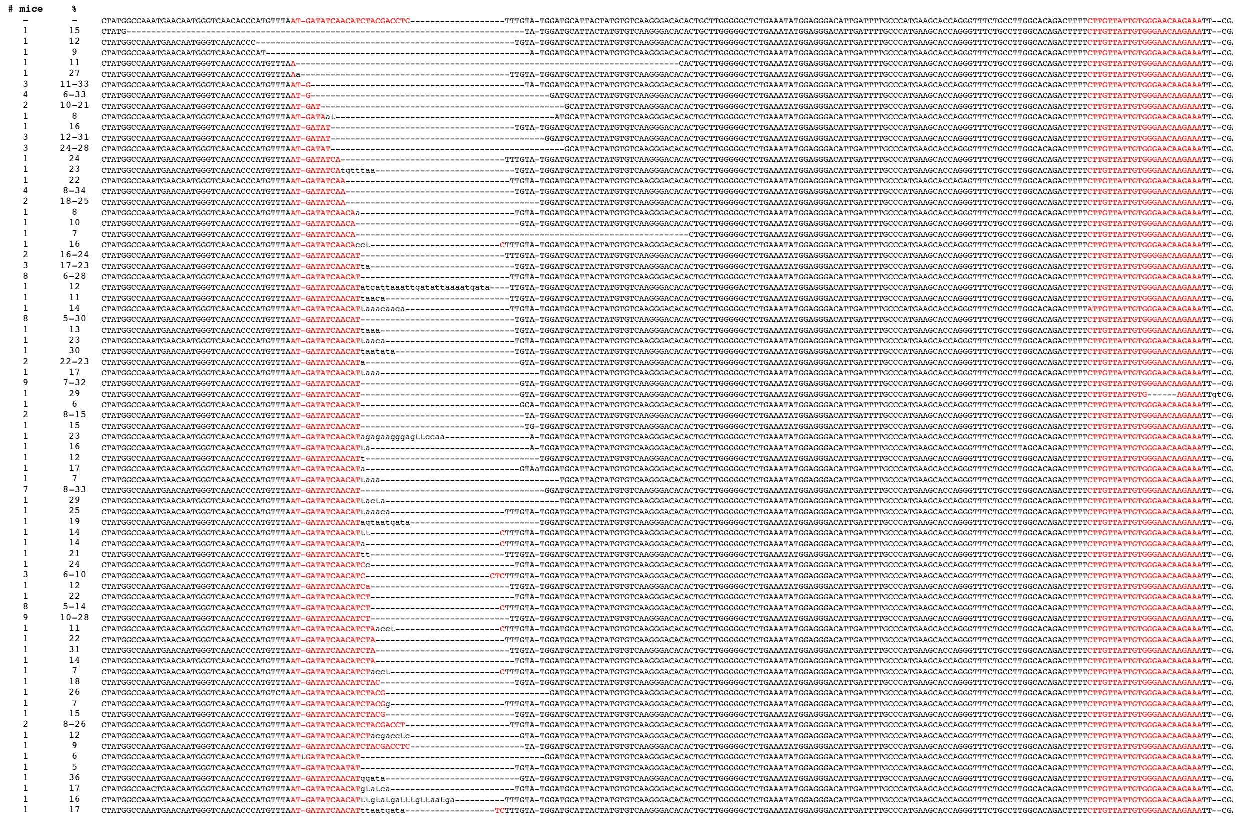

Figure S2 Sequence alignment for 74 alleles compared to the expected wild-type *Tyrosinase* sequence. The target site is indicated (red) along with deleted bases (-) and inserted or altered bases (lower case). Note that in some cases the exact breakpoint cannot be determined from the sequence data because bases on either side of the breakpoint are identical. For each allele, the number of founder mice observed carrying that allele is indicated along with the percentage of sequencing reads within the founder PCR product. For alleles observed in multiple founders, a range of percentages is given.
