# Supplementary material for: Highly Efficient Cpf1-Mediated Gene Targeting in Mice Following High Concentration Pronuclear Injection
Source: G3 (Bethesda). 2016 Dec 30;7(2):719–22. doi: 10.1534/g3.116.038091 (PMC5295614; doi:10.1534/g3.116.038091)
Supplement: Supplementary file 3 [file 719FigureS3.pdf]

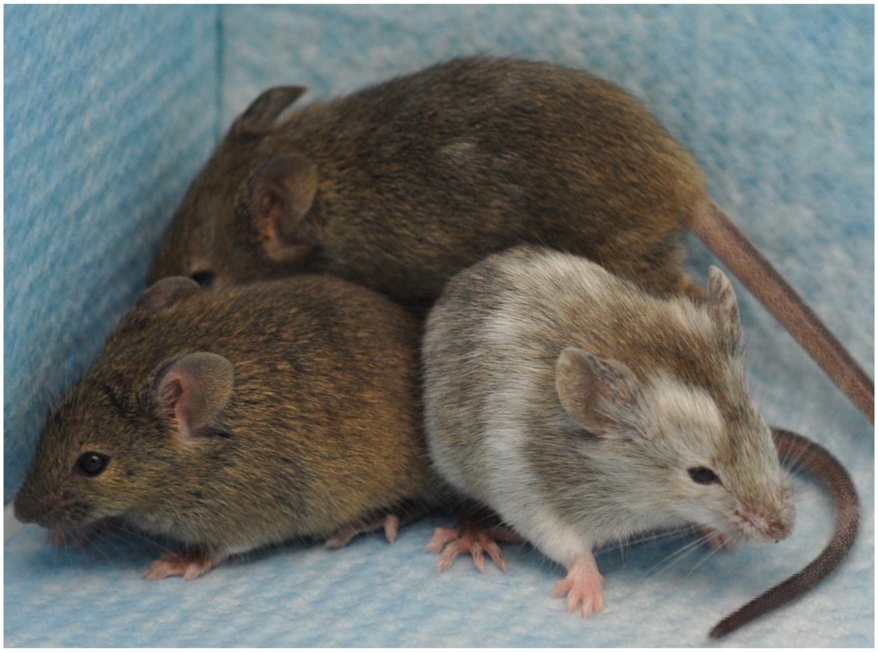

Figure S3 Mosaic transmission of AsCpf1-mediated mutant alleles. 6 founders were bred to confirm transmission of the mutant alleles: 1 albino mouse and 5 mosaics ranging from 10-70% albino coat color contribution. In crosses of all 6 mice to FVB, germline transmission of the mutant allele occurred. Two of the 6 mice are shown here: the mouse with the lowest albino coat color contribution (10%) in the center, and the mouse with the highest coat color contribution (70%) on the right. For comparison, a wild-type agouti mouse is shown on the left.
