# Supplementary material for: Highly Efficient Cpf1-Mediated Gene Targeting in Mice Following High Concentration Pronuclear Injection
Source: G3 (Bethesda). 2016 Dec 30;7(2):719–22. doi: 10.1534/g3.116.038091 (PMC5295614; doi:10.1534/g3.116.038091)
Supplement: Supplementary file 4 [file 719TableS1.pdf]

Table S1. Summary of injection sessions

Cpf1 (ng/µl)	Target A (ng/µl)	Target B (ng/µl)	Egg strain (♀ x ♂)	# eggs implanted	# pups or embryos recovered	# founders targeted	% founders targeted
10	2.5	2.5	B6xFVB	128	24	5/24	20.8%
10	2.5	2.5	B6xFVB	101	30	1/30	3.3%
10	2.5	2.5	FVBxB6	180	54	1/54	1.9%
10	2.5	2.5	FVBxB6	110	17	0/17	0.0%
10	2.5	2.5	FVBxB6	168	36	0/36	0.0%
10	2.5	-	FVBxB6	40	1	0/1	0.0%
10	2.5	-	FVBxB6	144	45	0/45	0.0%
50	100	100	B6xFVB	50	21	18/21	85.7%
50	100	100	B6xFVB	48	23	21/23	91.3%
50	100	100	B6 x B6	62	16	13/16	81.2%
50	100	100	FVBxB6	80	2	2/2	100%
50	100	-	FVBxB6	72	11	5/11	45.5%
50	100	-	FVBxB6	100	17	7/17	41.2%
**Average across all 12 injection sessions:**						**73/297**	**24.6%**
**Average for 7 low concentration injection sessions:**						**7/207**	**3.4%**
**Average for 6 high concentration injection sessions:**						**66/90**	**73.3%**
