# Supplementary material for: Highly Efficient Cpf1-Mediated Gene Targeting in Mice Following High Concentration Pronuclear Injection
Source: G3 (Bethesda). 2016 Dec 30;7(2):719–22. doi: 10.1534/g3.116.038091 (PMC5295614; doi:10.1534/g3.116.038091)
Supplement: Supplementary file 5 [file 719TableS2.pdf]

Table S2. Effect of strain on targeting rate for each injection concentration*

	B6 ♀ x FVB ♂	FVB ♀ x B6 ♂	Comparison (Fisher's)
**Total for all injection sessions**	45/98 (45.9%)	15/183 (8.2%)	**P < 0.0001**
**High concentration with 2 guides**	39/44 (88.6%)	2/2 (100%)	**P = 1.0000**
**High concentration with 1 guide**	-	12/28 (42.9%)	**-**
**Low concentration with 2 guides**	6/54 (11.1%)	1/107 (0.9%)	**P = 0.0060**
**Low concentration with 1 guide**	-	0/46 (0%)	**-**

***Data in this table is tallied from Supplemental Table 1**
