# Supplementary material for: Highly Efficient Cpf1-Mediated Gene Targeting in Mice Following High Concentration Pronuclear Injection
Source: G3 (Bethesda). 2016 Dec 30;7(2):719–22. doi: 10.1534/g3.116.038091 (PMC5295614; doi:10.1534/g3.116.038091)
Supplement: Supplementary file 6 [file 719TableS3.pdf]

Table S3. Effect of concentration on targeting rate for each strain*

	High Concentration	Low Concentration	Comparison (Fisher's)
**B6 ♀ x FVB ♂, all injections**	39/44 (88.6%)	6/54 (11.1%)	**$P < 0.0001$**
**B6 ♀ x FVB ♂ with 2 guides**	39/44 (88.6%)	6/54 (11.1%)	**$P < 0.0001$**
**B6 ♀ x FVB ♂ with 1 guide**	-	-	-
**FVB ♀ x B6 ♂, all injections**	14/30 (46.7%)	1/153 (0.7%)	**$P < 0.0001$**
**FVB ♀ x B6 ♂ with 2 guides**	2/2 (100%)	1/107 (0.9%)	**$P = 0.0005$**
**FVB ♀ x B6 ♂ with 1 guide**	12/28 (42.9%)	0/46 (0%)	**$P < 0.0001$**

***Data in this table is tallied from Supplemental Table 1**
