# Supplementary material for: Highly Efficient Cpf1-Mediated Gene Targeting in Mice Following High Concentration Pronuclear Injection
Source: G3 (Bethesda). 2016 Dec 30;7(2):719–22. doi: 10.1534/g3.116.038091 (PMC5295614; doi:10.1534/g3.116.038091)
Supplement: Supplementary file 7 [file 719TableS4.pdf]

Table S4: Comparison of successful targeting with AsCpf1

Reference	Delivery	Cpf1	Guide RNA	Gene	# of founders targeted	% of founders targeted
Hur et al. 2016	Pronuclear microinjection of RNPs	200 ng/µl protein	9000 ng/µl	Foxn1	10/12 blasts	83.0
Hur et al. 2016	Pronuclear microinjection of RNPs	200 ng/µl protein	9000 ng/µl	Foxn1	1/6 offspring	17.0
Hur et al. 2016	Electroporation of RNPs	100 ng/µl protein	250 ng/µl	Foxn1	16/25 blasts	64.0
Hur et al. 2016	Electroporation of RNPs	100 ng/µl protein	250 ng/µl	Foxn1	3/7 offspring	43.0
Hur et al. 2016	Electroporation of RNPs	100 ng/µl protein	250 ng/µl	Tyr	4/12 blasts	33.0
Hur et al. 2016	Electroporation of RNPs	100 ng/µl protein	250 ng/µl	Tyr	1/7 offspring	14.0
Kim et al. 2016	Cytoplasmic microinjection of RNA	50 ng/µl mRNA	100 ng/µl	p53 guide #1	12/16 embryos	75.0
Kim et al. 2016	Cytoplasmic microinjection of RNA	50 ng/µl mRNA	100 ng/µl	p53 guide #2	3/7 embryos	42.9
Kim et al. 2016	Cytoplasmic microinjection of RNA	50 ng/µl mRNA	100 ng/µl	p53 guide #1&2	19/24 newborns	79.2
Kim et al. 2016	Cytoplasmic microinjection of RNA	50 ng/µl mRNA	100 ng/µl	Prkdc guide #1& 2	2/11 newborns	18.2
Kim et al. 2016	Cytoplasmic microinjection of RNA	50 ng/µl mRNA	50 ng/µl	p53 & Prkdc (4 guides)	25/35 embryos	71.4
Watkins-Chow et al: "Low"	Pronuclear Injection of RNA	10 ng/µl mRNA	2.5 ng/µl	Tyr guide A & B	7/207 offspring& embryos	3.4*
Watkins-Chow et al: "High"	Pronuclear Injection of RNA	50 ng/µl mRNA	100 ng/µl	Tyr guide A & B	66/90 embryos	73.3*

*Results from the current publication are provided as an average of multiple injection sessions. See Supplemental Table 1 for details of individual injections. Results for Hur et al. 2016 and Kim et al. 2016 are presented as individual experiments as provided in publication for comparison.
